# The insulin-like growth factor-I receptor inhibitor figitumumab (CP-751,871) in combination with docetaxel in patients with advanced solid tumours: results of a phase Ib dose-escalation, open-label study

**DOI:** 10.1038/sj.bjc.6605767

**Published:** 2010-07-13

**Authors:** L R Molife, P C Fong, L Paccagnella, A H M Reid, H M Shaw, L Vidal, H-T Arkenau, V Karavasilis, T A Yap, D Olmos, J Spicer, S Postel-Vinay, D Yin, A Lipton, L Demers, K Leitzel, A Gualberto, J S de Bono

**Affiliations:** 1 The Royal Marsden NHS Foundation Trust/The Institute of Cancer Research, Sutton, Surrey, UK; 2 Pfizer Oncology, New London, CT, USA; 3 Penn State Hershey Medical Center, Hershey, PA, USA

**Keywords:** figitumumab (CP-751,871), insulin-like growth factor type 1 receptor (IGF-IR), chemosensitisation, monoclonal antibody, docetaxel

## Abstract

**Background:**

This phase Ib trial assessed safety, tolerability, and maximum tolerated dose (MTD) of figitumumab (CP-751,871), a fully human monoclonal antibody targeting the insulin-like growth factor type 1 receptor (IGF-IR), in combination with docetaxel.

**Methods:**

Patients with advanced solid tumours were treated with escalating dose levels of figitumumab plus 75 mg m^–2^ docetaxel every 21 days. Safety, efficacy, pharmacokinetics (PKs), and biomarker responses were evaluated.

**Results:**

In 46 patients, no dose-limiting toxicities were attributable to the treatment combination. Grade 3 and 4 toxicities included neutropaenia (*n*=28), febrile neutropaenia (*n*=11), fatigue (*n*=10), leukopaenia (*n*=7), diarrhoea (*n*=5), hyperglycaemia, lymphopaenia, cellulitis, DVT, and pain (all *n*=1). The MTD was not reached. Four partial responses were observed; 12 patients had disease stabilisation of ⩾6 months. Pharmacokinetic and biomarker analyses showed a dose-dependent increase in plasma exposure, and complete sIGF-IR downregulation at doses of ⩾3 mg kg^–1^. Pharmacokinetics of docetaxel in combination was similar to when given alone. Out of 18 castration-resistant prostate cancer patients, 10 (56%) had ⩾5 circulating tumour cells (CTCs) per 7.5 ml of blood at baseline: 6 out of 10 (60%) had a decline from ⩾5 to <5 CTCs and 9 out of 10 (90%) had a ⩾30% decline in CTCs after therapy.

**Conclusions:**

Figitumumab and docetaxel in combination are well tolerated. Further evaluation is warranted.

Alterations in the expression of components of the insulin-like growth factor (IGF) signalling pathway have been shown to have a critical role in the development of a variety of human malignancies, including lung, breast, prostate, thyroid, colorectal cancers, and sarcomas (Hankinson *et al*, 1998; Chang *et al*, 2002; Cardillo *et al*, 2003; Durai *et al*, 2005; Pollak, 2008). The IGF type 1 receptor (IGF-IR) pathway, initiated by the ligands IGF-1 and IGF-2, is associated with cellular mitogenesis, angiogenesis, tumour cell survival, and tumourigenesis in various tumour cell lines (Kalli *et al*, 2002; Kurmasheva and Houghton, 2006; Samani *et al*, 2007). Inhibition of IGF-IR in a range of tumour types has antiproliferative effects and synergises with other anticancer therapies, including cytotoxic chemotherapies (Benini *et al*, 2001; Cohen *et al*, 2005; Yin *et al*, 2005; Abe *et al*, 2006). Preclinical models have shown evidence of chemosensitisation of androgen-independent human prostate cancer cells when IGF-IR blockade was combined with either cisplatin, mitoxantrone, or paclitaxel (Hellawell *et al*, 2003). Inhibition of IGF-IR also enhanced docetaxel antitumour activity in animal models of castration-resistant prostate cancer (CRPC) (Cardillo *et al*, 2003), and synergised with trastuzumab in HER2^+^ breast cancer cells (Esparís-Ogando *et al*, 2008).

Figitumumab (CP-751,871) is a fully human IgG2 monoclonal antibody (mAb) highly specific for IGF-IR. Figitumumab blocks the binding of IGF-1 to IGF-IR, inhibits the downstream signalling activated by both IGF-1 and IGF-2, and induces prolonged receptor internalisation and degradation (Cohen *et al*, 2005). It inhibits the growth of tumour xenografts derived from colon (Colo-205), breast (MCF7), and lung (H460) cancer cell lines (Cohen *et al*, 2005). Additive tumour growth inhibition was observed when figitumumab was combined with adriamycin, 5-fluorouracil, or tamoxifen (Cohen *et al*, 2005), when compared with either of these cytotoxic therapies alone.

Figitumumab has been reported to be well tolerated in patients with solid tumours (Haluska *et al*, 2007) and myeloma (Lacy *et al*, 2008). The majority of adverse events were of grade 1 and 2, and included elevated transaminases, hyperglycaemia, anorexia and fatigue. Grade 3 hyperglycaemia was observed in one patient in the study of Lacy *et al* (2008), alongside grade 3 elevated aspartate aminotransferase (AST), whereas there was one episode each of grade 3 elevated *γ*-glutamyl transferase (GGT), arthralgia and fatigue in the study of Haluska *et al* (2007). No dose-limiting toxicities (DLTs) were observed in these phase I studies, in which the maximum tolerated dose (MTD) was not reached, and the maximal feasible dose and recommended phase II dose (RP2D) was 20 mg kg^–1^ every 3 or 4 weeks (Haluska *et al*, 2007; Lacy *et al*, 2008). On the basis of its ability to block IGF-IR and modulate chemosensitivity, the addition of figitumumab to docetaxel may improve the antitumour activity of single-agent docetaxel. This phase I dose-escalation trial was designed to determine the safety, tolerability, pharmacokinetic (PK), and pharmacodynamic (PD) effects of figitumumab given in combination with docetaxel in subjects with advanced solid tumours.

## Materials and methods

### Eligibility

Patients with histologic or cytologic confirmation of advanced solid tumours refractory to standard therapy were eligible. Other inclusion criteria included: age ⩾18 years; Eastern Cooperative Oncology Group performance status of 0 or 1; adequate bone marrow, renal, and hepatic function (absolute neutrophil count ⩾1500 *μ*l^–1^, haemoglobin ⩾10 g per 100 ml, platelets >100 000 *μ*l^–1^, creatinine clearance ⩾30 ml min^–1^, and total bilirubin equal to or less than the institution upper limit of normal (ULN), AST, and alanine aminotransferase (ALT) ⩽1.5 × ULN); and use of adequate contraception in patients with reproductive potential. Exclusion criteria included: anticancer therapy or surgery within 4 weeks, excluding luteinising hormone-releasing hormone (LHRH) analogues in prostate cancer patients; severe hypersensitivity reaction to docetaxel or drugs formulated in polysorbate 80; neuropathy of grade ⩾1; symptomatic or untreated brain metastases; pregnancy or lactation; significant active cardiac disease; concomitant high-dose corticosteroids (⩾100 mg prednisone per day or equivalent); serious active infection; and other uncontrolled significant medical illness.

The study protocol was approved by the ethics committee of the Royal Marsden Hospital and all patients gave written informed consent before any study procedures were performed. The study was conducted to Good Clinical Practice (GCP) specifications in accordance with the Declaration of Helsinki and its amendments.

### Study design and objectives

This was an open-label, phase Ib trial performed at a single centre. The primary objective was to define the RP2D of figitumumab when given in combination with docetaxel. Secondary objectives were to characterise the PK of figitumumab when given in combination with docetaxel, to evaluate the effect of figitumumab on the number of circulating tumour cells (CTCs) and their expression of IGF-IR (Allard *et al*, 2004; de Bono *et al*, 2007), to test for the occurrence of any antidrug antibody (ADA) response to figitumumab, to monitor the efficacy of figitumumab when given in combination with docetaxel, and to characterise the effect of figitumumab on docetaxel PK.

### Treatment

Figitumumab was administered intravenously (IV) after docetaxel (75 mg m^–2^) on day 1 every 21 days at doses of 0.1–20 mg kg^–1^ in dose-doubling cohorts of three to six patients. Dose reduction of docetaxel to 60 mg m^–2^ was permitted. In an expansion cohort to evaluate PK drug–drug interaction (DDI), in cycle 1 docetaxel was administered on day 1 followed by figitumumab on day 2; for subsequent cycles, both agents were administered on day 1. Patients were pre-medicated with oral dexamethasone 8 mg twice daily for 3 days starting at day –1. Patients continued treatment with the combination or single-agent figitumumab if docetaxel was discontinued, until disease progression or unacceptable toxicity was observed.

### Safety evaluation

Clinical and laboratory assessments for safety (physical examination, blood chemistry and haematology, urinalysis, adverse event, and concomitant medication queries) were performed before enrolment, before the next cycle, and at the end of treatment. Doppler echocardiograms were performed at baseline, end of cycles 1 and 6, end of study, and at follow-up (see below). Serum samples for monitoring ADA were collected from all patients at 30 min before dose in each cycle, at the end of study, and during follow-up visits. When figitumumab plasma concentrations were below the lower limit of quantification (LLOQ) of 120 ng ml^–1^, the ADA samples were analysed using a validated semiquantitative enzyme-linked immunosorbent assay (ELISA). In brief, the ADA samples were first incubated with figitumumab immobilised on a microtitre plate. After removal of unbound material by washing, anti-figitumumab antibodies were detected using biotinylated figitumumab, followed by addition of streptavidin-horseradish peroxidase conjugate and visualisation with 3,3′,5,5′-tetramethylbenzidine (TMB). The relative sensitivity of the assay was approximately 1 ng ml^–1^.

Toxicities were characterised according to the National Cancer Institute Common Terminology Criteria for Adverse Events (NCI-CTCAE), version 3.0. Radiologic (using Response Evaluation Criteria in Solid Tumours-1 (RECIST) guidelines) and biochemical evaluation of disease response was conducted every 6 weeks.

A DLT was defined as any one of the following adverse events occurring during cycle 1 if considered related to study treatment: (1) grade ⩾3 gastrointestinal toxicity despite the use of adequate medical intervention and/or prophylaxis; (2) any other grade ⩾3 toxicity not classified under CTCAE blood/bone marrow (except for grade 3 alopaecia and grade 3 *γ*-glutamyl transpeptidase); (3) grade 4 *γ*-glutamyl transpeptidase; (4) grade 4 neutropaenia (absolute neutrophil count <500 cells mm^–3^) persisting for ⩾7 consecutive days or complicated by fever (body temperature >38.0 °C or 100 °F) and requiring hospitalisation; (5) asymptomatic mitral thickening (>5 mm) with mitral regurgitation greater than mild or valve gradient >5 mm Hg on echocardiogram, as minor granulocytic and lymphocytic infiltration, oedema, and deposition of myxomatous material was observed in the mitral valve and subvalvular endocardium in preclinical testing; and (6) grade 4 thrombocytopaenia (platelets <25 000 cells mm^–3^). The use of colony-stimulating factors (G-CSF and GM-CSF) was permitted for the management of recurrent febrile neutropaenia.

### Evaluation of figitumumab and docetaxel PKs

For patients in the dose-escalation and expansion cohorts, blood samples for the evaluation of figitumumab PKs were collected before dose, and 1 h and 1, 3, and 7 days after figitumumab dose in cycles 1 and 4; predose in cycles 2, 3, 5, and beyond; and at the end of the study. For patients enroled into the PK DDI cohort, blood samples for evaluation of docetaxel PKs were collected in cycles 1 and 4 at pre-dose, 30, and 50 min after the start of, and 30 min and 1, 3, 8, and 24 h after the end of, docetaxel infusion. In addition, blood samples for determination of figitumumab concentrations were collected from patients enroled into the PK DDI cohort before dose in cycles 1–6 and 1 h after dose in cycle 4.

Plasma concentrations of figitumumab were analysed by a validated ELISA as previously described (Haluska *et al*, 2007). In brief, an IGF-1 soluble receptor extracellular domain was used in this assay to capture figitumumab. Figitumumab bound to the capturing receptor was then detected using horseradish peroxidase-conjugated mouse anti-human IgG2. The LLOQ for the assay was determined to be 120 ng ml^–1^. Plasma concentrations of docetaxel were determined using a validated high-performance liquid chromatography method coupled with tandem mass spectrometry (HPLC-MS/MS). The LLOQ of the HPLC-MS/MS assay was 10 ng ml^–1^.

Figitumumab and docetaxel plasma concentration–time data were analysed by noncompartmental methods (Gibaldi and Perrier, 1982) using WinNonlin version 3.2 (Pharsight, Mountain View, CA, USA). For treatment cycles with sufficient figitumumab PK data, area under the plasma concentration–time curve (AUC) from time 0 to the last sampling time point with quantifiable concentration within a cycle (AUC_last_) and from time 0 to the last day of a cycle (AUC_0–day22_) were determined using the linear/log trapezoidal approximation. The accumulation ratio of figitumumab was calculated as the ratio of cycle 4 AUC_0–day22_ to cycle 1 AUC_0–day22_. For docetaxel PK, the peak plasma concentration (*C*_max_) was determined by inspection of individual patient plasma concentration–time data. AUC_last_ values were determined using the linear/log trapezoidal approximation. For comparison of exposures of cycles 1 and 4, the *C*_max_ and AUC_last_ values were normalised to dose level.

### PD studies

Blood samples for the measurement of soluble (s)IGF-IR levels and the enumeration of CTCs, including IGF-IR-expressing CTCs, were collected from all patients on days 1 (before dosing of figitumumab) and 8 of each treatment cycle, and at the end of treatment. Total and IGF-IR-positive CTCs were isolated and enumerated using the CellTracks system (Immunicon, Huntingdon Valley, PA, USA) as previously described (de Bono *et al*, 2007). The sIGF-IR levels were determined using an enzyme immunoassay that detects the extracellular domain of the IGF-IR (Pollak *et al*, 2007; Pollak, 2008). The IGF-1R assay was validated and performed in the laboratory of the co-author Dr Laurence Demers. IGF-1R was determined in serum with a microtitre plate ELISA method (reagents obtained from R&D Systems, Minneapolis, MN, USA). The assay uses recombinant human IGF-1R for the standard, a mouse anti-human IGF-1R capture antibody, and a biotinylated detection antibody raised in goats. The mouse anti-human IGF-1R capture antibody showed <1% crossreactivity with IGF-1, IGF-II, and IGFBP 1-6. Assay sensitivity was 0.1 pg ml^–1^ and within-run imprecision was 5.3 and 7.1% at IGF-1R concentrations of 8.4 and 0.52 pg ml^–1^.

## Results

### Patient characteristics

A total of 46 patients with a median age of 59.4 years (range 25–79) were enroled (Table 1). The most common tumour types treated were CRPC (*n*=22, 47.8%) and oesophageal cancer (*n*=9, 19.6%). Of the 28 patients who had received previous chemotherapy, 3 had received at least one taxane-based regimen. Patients received a median of 4.5 courses of figitumumab (range 1–21) and a median of four courses of docetaxel (range 1–13). In all, 12 patients received ⩾10 cycles of figitumumab alone or with docetaxel. A dosing summary is provided in Table 2.

### Safety

Dose escalation of figitumumab proceeded safely from 0.1 to 20 mg kg^–1^ with no reported DLTs. One episode of grade 4 hyperglycaemia was observed during cycle 1 in a patient with metastatic oesophageal cancer and a history of type II diabetes mellitus treated with figitumumab at 20 mg kg^–1^. Another episode of grade 2 hyperglycaemia was reported. Other figitumumab-related toxicities are reported in Table 3 and include elevated ALT (*n*=5) and *γ*-glutamyl transferase (*n*=3), fatigue (*n*=3), nausea (*n*=3), and muscle spasms (*n*=3). Grade 3 and 4 toxicities related to docetaxel (Table 3) reflected the expected toxicity profile for this drug, and included neutropaenia (*n*=28), febrile neutropaenia (*n*=11), fatigue (*n*=10), leukopaenia (*n*=7), diarrhoea (*n*=5), lymphopaenia (*n*=1), cellulitis (*n*=1), deep-vein thrombosis (*n*=1), and pain (*n*=1). There were no mitral valve changes observed in serial echocardiograms as was the case in single-agent figitumumab studies. No patients required therapy with GCSF or GM-CSF. The MTD was not reached. Serum samples with figitumumab concentrations below the LLOQ were screened for ADA. Of the 60 ADA samples obtained from 17 patients, none was positive for ADA (<3.32). The RP2D was the maximum feasible dose of 20 mg kg^–1^ of figitumumab in combination with 75 mg m^–2^ of docetaxel.

### Pharmacokinetics

**Figure 1:**
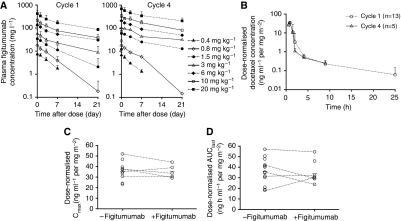
Pharmacokinetics of figitumumab and docetaxel. (**A**) Mean (±s.d.) plasma concentration–time profiles of figitumumab in cycles 1 and 4 when given in combination with docetaxel. (**B**) Dose-normalised docetaxel concentration–time profile in cycles 1 and 4. (**C**) Dose-normalised *C*_max_ in the absence and presence of figitumumab. (**D**) Dose-normalised AUC_last_ in the absence and presence of figitumumab (open circles indicate individual observations; short line indicates mean values; and dashed line joins observations in cycles 1 and 4 from the same patients). Abbreviations: −figitumumab, without figitumumab; +figitumumab, with figitumumab.

Plasma concentrations of figitumumab decreased in a multiexponential manner after an IV infusion (Figure 1A). The decline in concentration was more rapid at the lower concentration range than the higher concentration range. As shown in Table 4, the plasma concentration at the end of infusion (*C*_1 h_) and the AUC within a cycle (AUC_0–day22_) increased with dose for cycles 1 and 4. The increase in AUC_0–day22_ was approximately dose proportional. After repeated administration, there was a moderate accumulation in plasma exposure of figitumumab at dose levels of ⩾3 mg kg^–1^, with the mean accumulation ratio being approximately two-fold at 10 and 20 mg kg^–1^ (Table 4).

Evaluable docetaxel PK data acquired from the DDI expansion cohort were available from 13 subjects in cycle 1 (without figitumumab) and 5 subjects in cycle 4 (with figitumumab). The dose-normalised docetaxel PK profiles seemed to be similar between cycles 1 and 4. Figure 1B shows that in both cycles, docetaxel concentration increased during the 1-h infusion and decreased rapidly after the end of infusion. The dose-normalised docetaxel PK profiles seemed to be similar between cycles 1 and 4. The mean dose-normalised *C*_max_ (Figure 1C) and AUC_last_ (Figure 1D) of docetaxel in both cycles were also comparable. Of the five patients who had both cycles 1 and 4 docetaxel PK data, there was no systematic pattern of change in dose-normalised *C*_max_ and AUC_last_ between the two cycles. Overall, in the limited number of patients evaluated, figitumumab did not seem to considerably affect the PK of docetaxel.

### Pharmacodynamics

**Figure 2:**
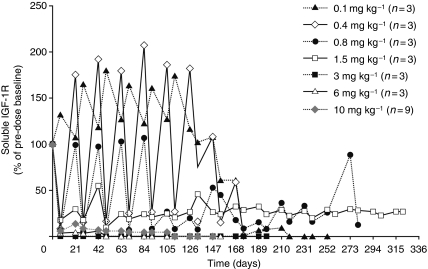
Mean concentration–time profiles of soluble IGF-IR after intravenous administration of figitumumab and docetaxel every 3 weeks. Concentrations of soluble IGF-IR were expressed as a percentage of individual pre-treatment baseline concentrations. Here, *N* indicates the number of patients with at least one measurement after the start of cycle 1 dosing.

All patients had detectable levels of sIGF-IR at study entry. Treatment with figitumumab resulted in a dose-dependent decrease in sIGF-IR, with higher doses translating to increasingly longer periods of serum-marker downregulation. At 1.5 and 3 mg kg^–1^ of figitumumab, complete sIGF-IR downregulation was achieved for the entire dosing period (Figure 2).

Circulating tumour cells ⩾5 per 7.5 ml of blood were enumerated in 15 patients, including 10 with CRPC. Of these 10 patients with CRPC, 60% (6 of 10) showed a fall from ⩾5 CTCs to <5 CTCs, and 80% (8 of 10) showed a ⩾30% fall in CTCs.

**Figure 3:**
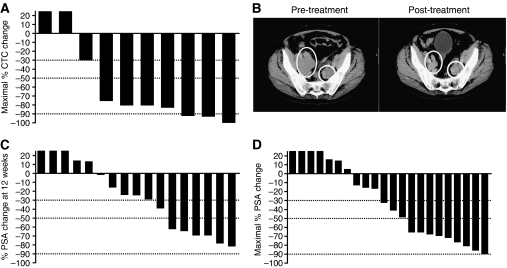
Efficacy of figitumumab and docetaxel in patients with CRPC. (**A**) Waterfall plot of maximal CTC declines in individual patients with baseline CTC count of ⩾5 per 7.5 ml, treated with figitumumab and docetaxel. Dotted lines indicate CTC declines of 30, 50, and 90%, respectively. (**B**) Radiologic response in a patient with metastatic disease to bilateral pelvic nodes. This patient also had extensive retroperitoneal (not shown) and bony metastases with a baseline PSA of 11 291 *μ*g l^–1^. Bilateral pelvic nodal metastatic deposits are indicated by circles at baseline (pre-treatment image). After four cycles of therapy, nodal disease showed a significant reduction in size (post-treatment image); PSA fell to a nadir of 1578 *μ*g l^–1^. The patient completed eight cycles of treatment with a maintained radiologic response, but with PSA progression and docetaxel-related fatigue. (**C**) Waterfall plot of PSA change from baseline to 12 weeks. (**D**) Waterfall plot of maximal PSA change for individual patients. Dotted lines indicate PSA declines of 30, 50, and 90%. Some patients had a PSA decline on study but this was short lived and PSA then rose again, explaining why the week 12 and maximal PSA declines are different. Abbreviations: CRPC, castration-resistant prostate cancer; CTC, circulating tumour cells; PSA, prostatic-specific antigen.

The maximal CTC fall for each of the 10 patients is shown in Figure 3A. Of the remaining five patients, two had gastric adenocarcinoma, two oesophageal adenocarcinoma, and one ovarian cancer. There was a CTC fall from ⩾5 per 7.5 ml to <5 in one of the two patients with gastric cancers (results not shown); the results of the patient with ovarian cancer were not evaluable. The results of IGF-IR CTCs have been reported elsewhere (de Bono *et al*, 2007).

### Efficacy

A total of 39 patients (including 18 CRPC patients) were evaluable for disease response. Four patients showed confirmed partial responses (PR): three with CRPC and one with oesophageal cancer. Figure 3B shows the response in pelvic nodal disease in a patient with metastatic CRPC to the bones and nodes. A fifth patient with CRPC showed an unconfirmed PR. All the radiologic responses were observed at doses of figitumumab >3 mg kg^–1^. In all, 12 patients had a best response of stable disease (SD), including eight with CRPC (SD range 6–16 months), one with chondrosarcoma (6 months), one with cervical cancer (7 months), one with gastric cancer (6 months), and one with oesophageal cancer (14 months). The remaining patients showed progressive disease.

Maximal prostate-specific antigen (PSA) declines of ⩾30, ⩾50, and ⩾90% were observed in 54% (12 of 22), 41% (9 of 22), and 5% (1 of 22) of patients, respectively. The increases or declines in PSA were confirmed with a second reading in a total of 17 patients and these were the patients evaluable for a PSA outcome. The percentage change in PSA from baseline to 12 weeks and maximal PSA decline at any point are depicted for each patient as waterfall plots in Figures 3C and D, respectively.

Of the six patients with CRPC whose CTCs fell from ⩾5 per 7.5 ml to <5 per 7.5 ml, two showed a radiological PR alongside ⩾50 and ⩾90% PSA decline, respectively. A third patient with nonmeasurable disease showed a ⩾50% PSA decline in the presence of a CTC fall. In the remaining three patients, two showed stable radiological disease and PSAs and one showed radiological and PSA PD. Of the four patients with adenocarcinomas of the upper gastrointestinal tract, three (two oesophageal and one gastric) showed a rise in CTCs from ⩾5 per 7.5 ml.

## Discussion

We have previously reported on the safety of single-agent figitumumab, a potent fully human mAb against a key factor in the IGF-1 system, IGF-IR (Haluska *et al*, 2007; Lacy *et al*, 2008). We now present our findings from a phase Ib study of patients with solid tumours using this mAb combined with the cytotoxic agent, docetaxel. The combination therapy was well tolerated and the previously reported figitumumab-related adverse events of hyperglycaemia and mild elevations in the liver transaminase enzymes were manageable (Haluska *et al*, 2007; Lacy *et al*, 2008). One patient with a past history of diabetes mellitus developed grade 4 hyperglycaemia. Steroid premedication and poor diabetic control were implicated as primary causes of this hyperglycaemic episode.

There was no apparent effect of figitumumab on the frequency or severity of observed neutropaenia. Although haematologic toxicity has been reported with other single-agent IGF-IR monoclonal antibodies such as MK-0646 and AMG-479 (Hidalgo *et al*, 2008; Tolcher *et al*, 2009), this toxicity does not seem to be significantly worsened when combined with chemotherapy, as found in our study and those of Sarantopoulos *et al* (2008) and Tolcher *et al* (2008). A preliminary report from a study by Tolcher *et al* (2008), in which AVE1642 (another mAb to IGF-IR) was combined with docetaxel in 14 patients, also reported no apparent exacerbation of docetaxel toxicity.

Increasing doses of figitumumab resulted in increased plasma concentrations of this antibody. The approximately two-fold accumulation in figitumumab plasma levels after dosing at ⩾10 mg kg^–1^ every 21 days confirmed previous findings that the dosing frequency of every 3 weeks is appropriate at these dose levels (Haluska *et al*, 2007). The PK exposure parameters (*C*_1 h_ and AUC_0–day22_) of figitumumab when combined with docetaxel were similar to those of single-agent figitumumab (Haluska *et al*, 2007), indicating that this combination does not considerably alter figitumumab PK parameters. Furthermore, in a limited number of patients, figitumumab did not seem to substantially affect the PKs of docetaxel. These results suggest that figitumumab at dose levels up to 20 mg kg^–1^ can be safely administered with docetaxel, with minimal docetaxel dose modification.

Administration of docetaxel and figitumumab in combination resulted in decreased sIGF-IR levels. At doses ⩾3 mg kg^–1^, there was a complete downregulation of sIGF-IR levels for the entire dosing cycle (Figure 2). This finding is consistent with the extended PK and PD properties of figitumumab previously reported (Haluska *et al*, 2007; Lacy *et al*, 2008).

It is interesting that sIGF-IR levels in patients receiving figitumumab at doses of 0.1–0.8 mg kg^–1^ resulted in levels higher than those observed at baseline, suggesting an intracellular feedback mechanism that can overcome the temporal lack of IGF-IR signalling. These preliminary data and those already published on the potential application of CTCs expressing IGF-IR (de Bono *et al*, 2007) support the ongoing analysis of PD end points, with a view to identifying predictive biomarkers of response to this and other agents in this class of drugs (Carden *et al*, 2009). In this study, IGF-IR+ CTCs were detected in all patients with ⩾5 CTCs per 7.5 ml at enrolment (de Bono *et al*, 2007). These patients had higher PSA levels than those patients who were IGF-IR CTC negative and were also more likely to show PSA declines of >50%. From this we suggested a potential for the use of IGF-IR positivity on CTCs as a molecular marker for identifying patients with CRPC who may benefit from anti-IGF-IR therapies (de Bono *et al*, 2007).

Although the demonstration of objective responses is not a key end point in phase I studies, clinical assessment of response to therapy in this study was of interest. Out of 22 patients with CRPC, 4 showed a confirmed PR, and 54, 41, and 5% of patients showed ⩾30, ⩾50, and ⩾90% falls in PSA, respectively, on therapy. In addition, a ⩾30% fall in CTC counts was observed in 80% of patients who had ⩾5 CTCs at baseline. Previous studies have shown that patients who convert from a CTC count ⩾5 at baseline to <5 after therapy had significantly better overall survival than those who did not (de Bono *et al*, 2008; Olmos *et al*, 2009). In addition, CTC counts were found to be an independent predictor of time to disease progression as well as survival. Half of the patients whose CTCs fell from ⩾5 to ⩽5 cells per 7.5 ml showed a radiological and/or PSA response; the numbers are small but this supports the use of CTCs as a biomarker of response. As a result of the activity observed in patients with CRPC, a randomised phase II study of figitumumab in combination with docetaxel and prednisone *vs* docetaxel and prednisone alone in patients with CRPC was initiated and is now close to completion.

A patient with oesophageal cancer completed a total of 18 courses of figitumumab (including an initial 10 courses of the treatment combination), achieving a PR after 4 cycles of the combination that was maintained until disease progression after 18 cycles. A second patient with oesophageal cancer completed 21 cycles of the antibody (including 10 with the combination) with a best response of SD, and complete resolution of tumour-associated dysphagia. This suggests that potentiation of the therapeutic effects of cytotoxic agents through a reversal of chemoresistance can lead to meaningful clinical outcomes. Phase II and III studies are ongoing to confirm efficacy in a number of tumour types, including non-small cell lung cancer (NSCLC), Ewing's sarcoma, gastrointestinal cancers, and breast cancer. Interestingly, although no clinical benefit was observed in the two patients with NSCLC in this study, significant clinical activity with the combination of figitumumab with paclitaxel and carboplatin over paclitaxel and carboplatin alone was observed in a randomised phase II study of 156 patients with NSCLC (Karp *et al*, 2009). In this trial, 54% of patients responded to the combination, compared with 42% of patients on paclitaxel and carboplatin alone. However, a randomised phase III study of this treatment combination was terminated in December 2009 as it was deemed unlikely to meet the primary end point of improved overall survival compared with chemotherapy alone. Further analysis of the data collected from this phase III study will determine whether it is possible to select patients who will likely benefit from this combination.

In conclusion, the combination of figitumumab at a maximum feasible dose of 20 mg kg^–1^ and docetaxel at 75 mg m^–2^ is safe and well tolerated in patients with advanced cancer, with no substantial alteration in the PKs of either agent. Randomised phase II and III studies of this, and other figitumumab treatment combinations, are ongoing in subjects with various solid tumours.

## Figures and Tables

**Table 1:** Baseline characteristics

**Characteristic**	***N*=46**
*Age, years*	
Median	59.4
Range	25–79
	
*Sex, n* (%)	
Male	40 (87)
Female	6 (13)
	
*ECOG PS, n* (%)	
0	9 (19.6)
1	37 (80.4)
	
*Tumour type, n* (%)	
CRPC	22 (47.8)
Oesophageal	9 (19.6)
GOJ	3 (6.5)
Sarcoma^a^	3 (6.5)
Gastric	2 (4.3)
Cervix	2 (4.3)
NSCLC	2 (4.3)
Vulva	2 (4.3)
Ovarian	1 (2.2)
	
*Previous therapy, n* (%)	
Surgery	31 (67.4)
Radiation	25 (54.3)
Chemotherapy	28 (60.9)
Hormonal	22 (47.8)
Other	10 (21.7)

Abbreviations: CRPC=castration-resistant prostate cancer; ECOG=Eastern Cooperative Oncology Group; GOJ=gastro-oesophageal junction; NSCLC=non-small cell lung cancer; PS=performance status.

a Includes two chondrosarcoma and one peripheral nerve sheath tumour.

**Table 2:** Treatment summary

		**No. of cycles**			
		**Docetaxel**		**Figitumumab**	
**Dose of figitumumab (mg** **kg^–1^)**	***n***	**Median**	**Range**	**Median**	**Range**
0.1	3	6	3–8	10	3–13
0.4	3	6	5–8	10	5–10
0.8	3	4	3–13	10	4–14
1.5	3	3	2–12	3	2–17
3	3	6	2–10	8	2–12
6	3	7	2–8	7	2–8
10	9	4	2–10	4	2–10
20	19	4	1–10	4	1–21

Docetaxel was dosed at 75 mg m^–2^.

**Table 3:** Treatment-related adverse events

**Toxicity**	**Grade**	**No. of patients (all cycles)**
*Figitumumab related*		
Elevated ALT	1/2	5
	3/4	0
Elevated GGT	1/2	3
	3/4	0
Fatigue	1/2	2
	3/4	1
Hyperglycaemia	1/2	1
	3/4	1
Muscle spasm	1/2	3
	3/4	0
Nausea	1/2	3
	3/4	0
*Docetaxel related*		
Alopaecia	1/2	3
	3/4	0
Anorexia	1/2	6
	3/4	0
Cellulitis	1/2	0
	3/4	1
Diarrhoea	1/2	8
	3/4	5
Deep-vein thrombosis	1/2	1
	3/4	1
Fatigue	1/2	16
	3/4	9
Febrile neutropaenia^a^	1/2	0
	3/4	11
Leukopaenia	1/2	3
	3/4	7
Lymphopaenia	1/2	2
	3/4	1
Mucositis	1/2	4
	3/4	0
Nausea	1/2	3
	3/4	0
Neutropaenia	1/2	4
	3/4	28
Pain	1/2	3
	3/4	1

Abbreviations: ALT=alanine aminotransferase; GGT=*γ*-glutamyl transferase.

a Includes neutropenic sepsis.

**Table 4:** Pharmacokinetic parameters (mean±s.d.) of figitumumab given in combination with docetaxel

	**Cycle 1**			**Cycle 4**				
**Dose (mg** **kg^–1^)**	***n***	***C***_**1** **h**_ **(mg** **l**^–1^**)**	**AUC_0–day22_ (mg** **h** **l**^–1^**)**	***n***	***C***_**1** **h**_ **(mg** **l**^–1^**)**	***C*_day22_ (mg** **l**^–1^**)**	**AUC_0–day22_ (mg** **h** **l**^–1^**)**	**Accumulation ratio**
0.1	3	1.34±0.36	—	2	1.04, 1.54	—	—	—
0.4	3	7.42±0.82	1010±164	3	6.9±1.75	—	774±40	0.78±0.16
0.8	3	17.9±6.8	2050±649	3	17.8±2.1	0.288^a^	2290±82	1.1±0.3
1.5	3	32.3±4.2	5110±2640	1	54.6	12.4	1260	1.5
3	3	57.7±23.7	10 500±3750	2	75.5, 126	31.4, 33.1	25 500, 24 600	2.2, 1.8
6	3	129±24	26 700±2770	2	203, 172	57.1, 37.2	46 400, 40 900	1.7, 1.4
10	9	211±33^b^	38 200±8000	7	324±48^c^	57.6, 101^d^	72 400±18 900	1.8±0.4
20	6	407±160^e^	82 100±23 500	5	658±185	199±73^f^	158 000±64 300	2.0±0.7

Abbreviations: AUC_0–day22_=area under the plasma concentration–time curve from time 0 to day 22; C_1 h_=plasma concentration at 1 h after the end of infusion; C_day22_=plasma concentration at day 22 of the cycle.

*n* indicates the number of patients included in the analysis.

a *n*=1.

b *n*=8.

c *n*=6.

d *n*=2.

e *n*=5.

f *n*=4.
